# Generalized anxiety disorder, psychiatric comorbidities, and suicide

**DOI:** 10.1007/s00127-025-02985-2

**Published:** 2025-08-26

**Authors:** Li-Chi Chen, Wei-Sheng Huang, Shih-Jen Tsai, Chih-Ming Cheng, Wen-Han Chang, Ya-Mei Bai, Tung-Ping Su, Tzeng-Ji Chen, Mu-Hong Chen

**Affiliations:** 1 Department of Psychiatry, Taipei Veterans General Hospital, No. 201, Shih-Pai Road, Sec. 2, Taipei, 11217, 28344012 Taiwan; 2 Department of Psychiatry, College of Medicine, National Yang Ming Chiao Tung University, Taipei, Taiwan; 3 Department of Family Medicine, Taipei Veterans General Hospital, Taipei, Taiwan; 4 Institute of Hospital and Health Care Administration, National Yang Ming Chiao Tung University, Taipei, Taiwan; 5 Department of Psychiatry, General Cheng Hsin Hospital, Taipei, Taiwan; 6 Department of Family Medicine, Hsinchu Branch, Taipei Veterans General Hospital, Hsinchu, Taiwan

**Keywords:** Generalized anxiety disorder, Suicide, Psychiatric comorbidities, Taiwan

## Abstract

**Background:**

Several studies have suggested a positive association between generalized anxiety disorder (GAD) and suicidal symptoms, particularly suicidal thoughts. Nevertheless, whether GAD is an independent risk factor for subsequent suicide is poorly understood.

**Methods:**

Analyzing data on the entire Taiwanese population (*N* = 29,077,426), we followed 322,855 patients with GAD and 1,291,420 individuals without GAD matched for birth date and sex over the period from 2003 to 2017. Deaths by suicide were confirmed using Taiwan’s Database of All-cause Mortality. Psychiatric disorders comorbid with GAD were also assessed, specifically schizophrenia, bipolar disorder, major depression, alcohol use disorder, substance use disorders, obsessive-compulsive disorder, and neurodevelopmental disorders.

**Results:**

A total of 2,051 (0.64%) individuals died by suicide in the GAD group, and 1,378 (0.11%) died by suicide in the control group. Cox regression models with adjustments for demographic characteristics, psychiatric comorbidities, and Charlson Comorbidity Index scores demonstrated that both men (hazard ratio [HR]: 2.64, 95% confidence interval [CI]: [2.29, 3.05]) and women (HR: 2.70, 95% CI: [2.33, 3.13]) with GAD were more likely to die by suicide than individuals in the control group

**Discussion:**

GAD was a risk factor for death by suicide when controlling for various sociodemographic and clinical factors,including comorbid schizophrenia, bipolar disorder, major depressive disorder, and alcohol and substance use disorders.Suicide prevention strategies must be developed for individuals with GAD and associated psychiatric comorbidities.

Generalized anxiety disorder (GAD) is a common and disabling mental disorder characterized by persistent, excessive, and unrealistic worry about everyday things. Individuals with GAD also have various nonspecific psychological and physical symptoms, such as fatigue, muscle tension, difficulties with concentration, and irritability [1, 2]. The Global Burden of Disease study estimated that 4.05% of individuals globally, or 301 million people, have anxiety disorders and that the prevalence of these disorders has increased by more than 50% from 1990 to 2019 [3]. Furthermore, anxiety disorders were associated with 44.5 million disability-adjusted life-years globally in 2020, a statistic for mental disorders only surpassed by the 49.4 million disability-adjusted life-years associated with major depressive disorder [4].

At least one study has revealed an association between GAD and suicidal symptoms, including suicidal thoughts, suicide attempts, and death by suicide [5]. The Netherlands Mental Health Survey and Incidence Study demonstrated that patients with GAD were more likely to have suicidal thoughts (odds ratio [OR]: 4.23, 95% confidence interval [CI]: [1.96, 9.16]) and attempt suicide (OR: 5.24, 95% CI: [2.10, 13.06]) compared with those without GAD [5]. However, the ORs decreased to 2.48 and 2.35, respectively, after adjusting for comorbid psychiatric disorders, such as schizophrenia, bipolar disorder, depression, alcohol use disorders (AUD), and substance use disorders (SUD) [5]. Additionally, Khan et al. analyzed data from the 2002 United States Food and Drug Administration database and determined that the annual suicide rate was 193/100,000 for patients with GAD, far higher than that for the general population [6]. A meta-analysis of 852,159 participants (52.2% of them with anxiety disorders) revealed that anxiety disorders were associated with suicidal ideation (OR: 1.49, 95% CI: [1.18, 1.88]) and suicide attempts (OR: 1.64, 95% CI: [1.47, 1.83]), but not with deaths from suicide (OR: 1.01, 95% CI [0.87, 1.18]) [7]. Moreover, prior research indicated that patients with GAD were significantly related to increased psychosocial issues, which enhanced the vulnerability of patients to stressful situations, including suicide risk [8].

However, the incidence of psychiatric comorbidities commonly associated with anxiety disorders, especially schizophrenia and major affective disorders, renders the definitive determination of GAD as an independent risk factor for suicide [5]. In addition, evidence has shown a significant relationship between treatment nonadherence and elevated suicide risk in patients with major affective disorders [9]. The presence of comorbid AUD and SUD may further compound these risks, intensifying both nonadherence and suicidality [9]. Furthermore, the aforementioned studies regarding an association between GAD and suicide have been conducted in Western countries, which may limit their generalizability to individuals from other populations, such as those in Asia.

The present study utilized data from Taiwan’s National Health Insurance Research Database (NHIRD), which contains data on the entire population of Taiwan (*N* = 29,077,426), and a longitudinal follow-up study design (following individuals for the period from 2003 to 2017) to investigate the independent association of GAD with suicide after controlling for major psychiatric comorbidities. We hypothesized that, regardless of the presence of schizophrenia and major affective disorders or AUD and SUD, individuals with GAD would have a higher suicide risk during the follow-up period than individuals without GAD.

## Methods

### Data source

The NHIRD, which contains detailed health-care data on more than 99.7% of Taiwan’s population, is audited and made available for research purposes by the Ministry of Health and Welfare’s Taiwan Health and Welfare Data Science Center. To protect patient privacy, individual medical records are anonymous in the NHIRD. For the purpose of analyzing the suicide risk among people with GAD, we linked the Longitudinal Health Insurance Database of the NHIRD, which contains all medical records from 2003 to 2017 of the entire Taiwanese population, and the Database of All-cause Mortality, which includes all-cause mortality records from 2003 to 2017 of the entire Taiwanese population. Taiwanese clinical practice uses the International Classification of Diseases, Ninth or Tenth Revision, Clinical Modification (ICD-9-CM [2003–2014] or ICD-10-CM [2015–2017]). The institutional review board of Taipei Veterans General Hospital approved the study protocol and waived the requirement for informed consent because deidentified data were used in this study and no participants were actively enrolled. The NHIRD has been used in numerous epidemiological studies in Taiwan [10–13].

### Inclusion criteria for individuals with GAD and the control group

**Fig. 1:**
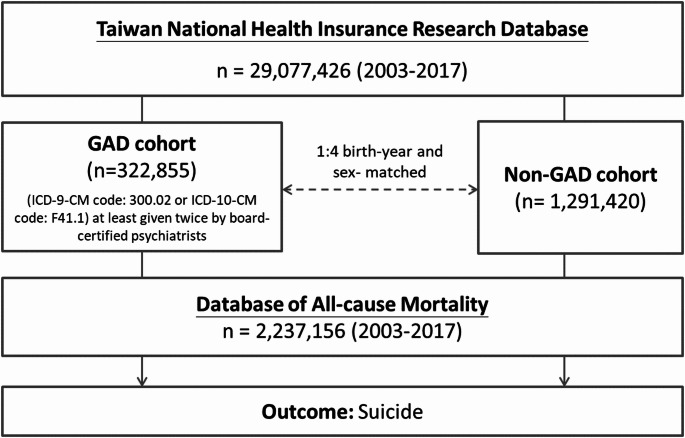
Study flowchart.GAD: generalized anxiety disorder

The GAD group consisted of people who were diagnosed with GAD (ICD-9-CM code: 300.02 or ICD-10-CM code: F41.1) at least twice by board-certified psychiatrists over the entire observation period (Fig. 1). To reduce the confounding effects of age and sex, a 1:4 case–control matched analysis was conducted based on birth year and sex. After excluding all individuals who had ever received a diagnosis of GAD anytime from the database, a random selection was made from the entire Taiwanese population to form the control group (Fig. 1). The urbanization level of residence (levels 1–4, most to least urbanized) was assessed as a proxy for health-care availability in Taiwan [14]. The GAD diagnosis was regarded as a time-dependent variable. Suicide was identified between 2003 and 2017 from the Database of All-cause Mortality. The Charlson Comorbidity Index (CCI) scores for patients with GAD and matched controls were computed. To ascertain the systemic health status of every enrolled subject, the CCI, which consists of 22 physical conditions, was also evaluated from 2003 to 2017 (or mortality) [15]. Furthermore, because GAD may be comorbid with schizophrenia (ICD-9-CM code: 295 or ICD-10-CM code: F20, F25), bipolar disorder (ICD-9-CM codes: 296 except 296.2, 296.3, 296.9, and 296.82 or ICD-10-CM codes: F30, F31), major depressive disorder (ICD-9-CM codes: 296.2, 296.3, 300.3, 311 or ICD-10-CM codes: F32, F33, F34), OCD (ICD-9-CM code: 300.3 or ICD-10-CM code: F42), attention-deficit hyperactivity disorder (ADHD) (ICD-9-CM code: 314 or ICD-10-CM code: F90), autism spectrum disorder (ASD) (ICD-9-CM codes: 299.0, 299.8, 299.9 or ICD-10-CM codes: F84.0, F84.5, F84.8, F84.9), AUD (ICD-9-CM codes: 291, 303.0, 303.9, 305.0 or ICD-10-CM code: F10), and SUD (ICD-9-CM codes: 292, 304, 305 except 305.0 and 305.1 or ICD-10-CM codes: F11, F12, F13, F14, F15, F16, F18, F19), which are also associated with suicide risk [16–18], these major psychiatric comorbidities also were investigated from 2003 to 2017 (or mortality) for further evaluation of the effect of comorbidities on the risk of suicide. These psychiatric disorders were diagnosed at least twice by board-certified psychiatrists.

### Statistical analysis

We used repeated measure analyses of variance with the general linear models for continuous variables and conditional logistic regressions for nominal variables for between-group comparisons in grouping data. Time-dependent Cox regression models with adjustment for sex, birth year, income, level of urbanization, psychiatric comorbidities and CCI were used to calculate HR and 95% CI of subsequent suicide between groups. Using Cox regression models with adjustments for sex, birth year, income, levels of urbanization, and CCI, we examined the effects of psychiatric comorbidities, such as schizophrenia, bipolar disorder, major depressive disorder, ASD, ADHD, AUD, and SUD, on the suicide risk among individuals with GAD in comparison to the control group. he proportional hazards assumptions were verified using the log-minus-log plots, resulting in no considerable violation. A two-tailed *p* value of < 0.05 was considered statistically significant. All data processing and statistical analyses were performed using the Statistical Analysis Software Version 9.1 (SAS Institute, Cary, NC, USA).

## Results

Overall, 322,855 patients with GAD and 1,291,420 birthdate-/sex-matched individuals without GAD were included in the present study, with a female predominance (61.1%) (Table 1). Individuals in the GAD group were more likely to have a higher income (*p* < 0.001), reside in an urban region (*p* < 0.001), and have higher CCI scores (*p* < 0.001) than those in the control group (Table 1). In addition, of all patients with GAD, 9463 (2.93%) individuals were comorbid with schizophrenia, 22,144 (6.86%) with bipolar disorder, 176,951 (54.81%) with major depressive disorder, 10,570 (3.27%) with OCD, 656 (0.20%) with ASD, 4201 (1.30%) with ADHD, 9279 (2.87%) with AUD, and 10,911 (3.38%) with SUD (Table 1).

**Table 1:** Demographic characteristics of patients with GAD and matched controls

	Patients with GAD *n* = 322,855	Comparison group *n* = 1,291,420	*p*-value
Birth Year (n, %)			> 0.999
−1950	67,224 (20.82)	268,896 (20.82)	
1951–1960	72,321 (22.40)	289,284 (22.40)	
1961–1970	71,216 (22.06)	284,864 (22.06)	
1971–1980	59,353 (18.38)	237,412 (18.38)	
1981–1990	37,525 (11.62)	150,100 (11.62)	
1991–2000	13,735 (4.25)	54,940 (4.25)	
2000-	1481 (0.46)	5924 (0.46)	
Male (n, %)	125,582 (38.90)	502,328 (38.90)	> 0.999
Monthly Income (n, %)			< 0.001
0-1000 USD	107,458 (33.28)	478,461 (37.05)	< 0.001
1001–1800 USD	135,150 (41.86)	522,356 (40.45)	< 0.001
≥ 1801 USD	80,247 (24.86)	290,603 (22.50)	< 0.001
Level of urbanization (n, %)			< 0.001
1 (Urban)	73,740 (22.84)	258,157 (19.99)	< 0.001
2	88,244 (27.33)	328,268 (25.42)	< 0.001
3	57,234 (17.73)	236,241 (18.29)	< 0.001
4	68,152 (21.11)	278,446 (21.56)	< 0.001
5 (Rural)	35,485 (10.99)	190,308 (14.74)	< 0.001
CCI (n, %)			< 0.001
0	44,346 (13.74)	416,892 (32.28)	< 0.001
1–2	119,464 (37.00)	474,120 (36.71)	< 0.001
> 2	159,045 (49.26)	400,408 (31.01)	< 0.001
Psychiatric comorbidities (n, %)			
Schizophrenia	9463 (2.93)	11,216 (0.87)	< 0.001
Bipolar disorders	22,144 (6.86)	9006 (0.70)	< 0.001
Major depressive disorder	176,951 (54.81)	65,809 (5.10)	< 0.001
OCD	10,570 (3.27)	2482 (0.19)	< 0.001
ASD	656 (0.20)	563 (0.04)	< 0.001
ADHD	4201 (1.30)	2247 (0.17)	< 0.001
AUD	9279 (2.87)	5667 (0.44)	< 0.001
SUD	10,911 (3.38)	5139 (0.40)	< 0.001

GAD: generalized anxiety disorder; OCD: obsessive compulsive disorder; USD: United State dollar; CCI: Charlson Comorbidity Index; ASD: autism spectrum disorder; ADHD: attention deficit hyperactivity disorder; AUD: alcohol use disorder; SUD: substance use disorder

Cox regression models with adjustment for demographic characteristics, psychiatric comorbidities, and CCI demonstrated that both males (HR: 2.64, 95% CI: [2.29, 3.05]) and females (2.70, [2.33, 3.13]) with GAD were more likely to die by suicide compared with the control group (Table 2). Table 3 showed the comorbid effects of additional psychiatric disorders with GAD on suicide risk. Major depressive disorder had the highest comorbid effect on suicide (HR: 7.41, 95% CI: [6.83, 8.03]) among individuals with GAD, followed by bipolar disorder (4.18, [3.63, 4.8]), schizophrenia (3.85, [3.23, 4.59]), AUD (3.79, [3.17, 4.52]), SUD (3.76, [3.18, 4.44]), and OCD (2.55, [2.06, 3.16]) (Table 3). Individuals with GAD with and without ADHD (HR: 2.00, 95% CI: [1.26, 3.17] vs. 2.45, [2.23, 2.68]) had similar suicide risk (Table 1). Owing to the limited cases with dual diagnoses of GAD and ASD, only those with GAD without ASD (HR: 2.45, 95% CI: [2.24, 2.68]) had an increased risk of suicide (Table 3).

**Table 2:** Suicide risk between patients with GAD and matched controls

	All sample			Male sample			Female sample		
	Event, n (%)	Suicide rate (per 100,000 person-year)	HR# (95% CI)	Event, n (%)	Suicide rate (per 100,000 person-year)	HR# (95% CI)	Event (n)	Suicide rate (per 100,000 person-year)	HR# (95% CI)
Patients with GAD	2051 (0.64)	43.06	**2.65 (2.39–2.94)**	972 (0.77)	52.75	**2.64 (2.29–3.05)**	1079 (0.55)	36.95	**2.70 (2.33–3.13)**
Comparison group	1378 (0.11)	7.21	1 (ref.)	778 (0.15)	10.51	1 (ref.)	600 (0.08)	5.13	1 (ref.)

GAD: generalized anxiety disorder; HR: hazard ratio; CI: confidence interval; CCI: Charlson Comorbidity Index

# adjusting for sex, birth year, income, level of urbanization, psychiatric comorbidities and CCI

Bold type indicates the statistical significance

**Table 3:** Suicide risk between patients with GAD with different psychiatric comorbidities and matched controls.–

	Suicide (HR, 95%)^#^		
	All sample	Male sample	Female sample
Comparison group	1.00 (ref.)	1.00 (ref.)	1.00 (ref.)
Patients with GAD			
Without schizophrenia	**2.36 (2.15–2.59)**	**2.29 (2.01–2.61)**	**2.43 (2.13–2.77)**
With schizophrenia	**3.85 (3.23–4.59)**	**3.81 (2.98–4.87)**	**4.07 (3.17–5.22)**
Comparison group	1.00 (ref.)	1.00 (ref.)	1.00 (ref.)
Patients with GAD			
Without bipolar disorder	**2.37 (2.16–2.60)**	**2.34 (2.06–2.67)**	**2.39 (2.09–2.73)**
With bipolar disorder	**4.18 (3.63–4.8)**	**3.38 (2.74–4.19)**	**4.70 (3.89–5.68)**
Comparison group	1.00 (ref.)	1.00 (ref.)	1.00 (ref.)
Patients with GAD			
Without major depressive disorder	**2.89 (2.59–3.22)**	**2.69 (2.32–3.11)**	**3.16 (2.68–3.73)**
With major depressive disorder	**7.41 (6.83–8.03)**	**6.41 (5.71–7.18)**	**8.53 (7.61–9.57)**
Comparison group	1.00 (ref.)	1.00 (ref.)	1.00 (ref.)
Patients with GAD			
Without OCD	**2.44 (2.23–2.68)**	**2.37 (2.08–2.69)**	**2.52 (2.21–2.87)**
With OCD	**2.55 (2.06–3.16)**	**2.54 (1.88–3.43)**	**2.66 (1.96–3.61)**
Comparison group	1.00 (ref.)	1.00 (ref.)	1.00 (ref.)
Patients with GAD			
Without ASD	**2.45 (2.24–2.68)**	**2.38 (2.09–2.70)**	**2.53 (2.22–2.88)**
With ASD	1.24 (0.31–5.02)	1.88 (0.46–7.65)	n.p.
Comparison group	1.00 (ref.)	1.00 (ref.)	1.00 (ref.)
Patients with GAD			
Without ADHD	**2.45 (2.23–2.68)**	**2.38 (2.09–2.70)**	**2.53 (2.22–2.88)**
With ADHD	**2.00 (1.26–3.17)**	**2.05 (1.14–3.67)**	**2.22 (1.04–4.71)**
Comparison group	1.00 (ref.)	1.00 (ref.)	1.00 (ref.)
Patients with GAD			
Without AUD	**2.39 (2.18–2.62)**	**2.30 (2.02–2.61)**	**2.48 (2.18–2.83)**
With AUD	**3.79 (3.17–4.52)**	**3.66 (2.92–4.58)**	**4.46 (3.33–5.97)**
Comparison group	1.00 (ref.)	1.00 (ref.)	1.00 (ref.)
Patients with GAD			
Without SUD	**2.41 (2.2–2.64)**	**2.34 (2.06–2.67)**	**2.48 (2.17–2.82)**
With SUD	**3.76 (3.18–4.44)**	**3.39 (2.71–4.25)**	**4.51 (3.51–5.78)**

GAD: generalized anxiety disorder; OCD: obsessive compulsive disorder; ASD: autism spectrum disorder; ADHD: attention deficit hyperactivity disorder; AUD: alcohol use disorder; SUD: substance use disorder

# Separate Cox regression models with adjustment of sex, birth year, income, level of urbanization, and CCI

**Bold type** indicates the statistical significance

## Discussion

Our findings support the hypothesis that GAD is an independent risk factor for death by suicide after adjusting for comorbid psychiatric disorders, specifically schizophrenia, bipolar disorder, major depressive disorder, OCD, neurodevelopmental disorders, AUD, and SUD. Additionally, we observed an additive effect of major psychiatric comorbidities with GAD, especially schizophrenia, bipolar disorder, major depressive disorder, AUD, and SUD, on suicide. Individuals comorbid with major depressive disorder and GAD exhibited the highest suicide risk (up to seven times higher than that for the non-GAD controls), followed by those comorbid with bipolar disorder, schizophrenia, AUD, SUD, and OCD.

At least one epidemiological study revealed an association between GAD and suicide, regardless of comorbidity with other major psychiatric disorders, such as schizophrenia and major affective disorders [5]. A national study of 887,859 veterans with depression in the United States observed a significant association between GAD and death by suicide (OR: 1.27, 95% CI: [1.09, 1.47]) [19], suggesting that GAD may be a precipitating factor of suicide in patients with depression. However, Bentley et al. suggested that anxiety disorders are associated with suicidal thoughts (OR: 1.49, 95% CI: [1.18, 1.88]) and suicide attempts (OR: 1.64, 95% CI: [1.47–1.83]), but not death by suicide (OR: 1.01, 95% CI: [0.87, 1.18]) [7]. Sareen et al. followed 4,796 adult participants (52.4% of them with anxiety disorders) for 3 years and observed that GAD was associated with suicidal thoughts (OR: 1.96, 95% CI: [1.45, 2.64]) and suicide attempts (OR: 1.33, 95% CI: [0.86, 2.05]) at baseline, independent of major psychiatric disorders (schizophrenia, mood disorders, AUD, and SUD), revealing that GAD independently predicted the occurrence of suicidal thoughts (OR: 2.48, 95% CI: [1.02, 6.06]) and suicide attempts (OR: 2.35, 95% CI: [0.78, 7.10]) during follow-up [5].

Sareen et al. also identified a similar influence of anxiety disorders alone (OR: 3.34, 95% CI: [1.75, 6.40]) and mood disorders alone (OR: 3.46, 95% CI: [1.78, 6.72]) on new-onset suicidal thoughts during the 3-year follow-up [5], consistent with our findings that a diagnosis of GAD alone was associated with death by suicide and that psychiatric comorbidities with GAD, such as schizophrenia, bipolar disorder, major depressive disorder, AUD, and SUD, increased the likelihood of death by suicide. Furthermore, the nonoverlapping CI between the group with GAD only and the group with GAD and psychiatric comorbidities (schizophrenia, bipolar disorder, major depressive disorder, AUD, and SUD) in the present study supports the hypothesis that GAD is an independent risk factor for death by suicide, suggesting that individuals with GAD and its psychiatric comorbidities should be closely monitored for the development of suicidal ideation. However, we observed no additive comorbid effect of OCD, ASD, or ADHD on suicide among patients with GAD, a finding attributable to the small sample sizes of the groups with comorbid ASD and ADHD and the overlapping factor of anxiety between GAD and OCD.

Dysregulation of the hypothalamic–pituitary–adrenal (HPA) axis may be the biological cause of the association between GAD and suicide [20, 21]. In this context, Lenze et al. analyzed the salivary cortisol levels of patients with GAD at six daily time points (waking, waking + 30 min, noon, afternoon, evening, and bedtime) and revealed that patients with GAD exhibited higher levels of both peak and total cortisol than the control group. Additionally, they observed a positive correlation between cortisol levels and GAD severity as measured by the GAD Severity Scale [22]. Furthermore, they suggested that 12-week treatment with escitalopram reduced salivary cortisol levels and improved anxiety symptoms [23]. Moreover, Vreeburg et al. reported that current anxiety disorders were associated with higher cortisol levels on awakening (*p* = 0.002) and also observed a trend toward higher morning cortisol levels (*p* = 0.08) in individuals whose anxiety disorders were in remission [24]. Additionally, a meta-analysis of 779 individuals who had attempted suicide and 1,447 individuals who had not attempted suicide uncovered a positive association between cortisol levels and suicide attempts, especially among individuals aged < 40 years [20]. Another study that evaluated the postmortem brains of 24 individuals who died by suicide and 24 normal controls discovered increased mRNA levels of corticotropin-releasing factor in the prefrontal cortex (PFC) and the central nucleus of the amygdala in those who died by suicide compared with the controls [25]. Finally, Pandey et al. demonstrated a substantial decrease in the protein and gene expression of glucocorticoid receptors in the PFC and amygdala of the postmortem brains of individuals who died by suicide [26].

This study has several limitations. First, we may have underestimated the prevalence of GAD in the Taiwanese population because only those who sought medical consultation and help were identified in the NHIRD. However, all GAD diagnoses were made by board-certified psychiatrists, improving the diagnostic validity in those individuals we studied. Second, time-dependent Cox regression models with adjustment for demographic data, psychiatric comorbidities and CCI were used to examine suicide risk between groups in our study. The analyses of dying from other causes as a competing risk were not performed in the present study because suicide always occurred much earlier than other mortality causes, especially natural death. Third, the NHIRD lacks information on environmental factors, psychosocial stress, lifestyle, and other variables that could influence the study’s outcomes. Therefore, we could not comprehensively investigate the contributions of these factors to suicide risk.

In conclusion, the present study observed that, compared with individuals who did not have GAD, patients with GAD were more likely to die by suicide during the follow-up period (2003–2017) after adjusting for psychiatric comorbidities, specifically schizophrenia, bipolar disorder, major depressive disorder, neurodevelopmental disorders, AUD, and SUD. GAD was individually significantly associated with the risk of suicide, and having additional psychiatric comorbidities with GAD increased this risk. Identifying GAD comorbidity is clinically relevant and important for suicide prevention in patients with severe mental disorders. To mitigate the risk of suicide in individuals with GAD, suicide prevention strategies targeting individuals with GAD and related psychiatric comorbidities should be developed and implemented. Further studies are required to clarify the pathomechanisms underlying anxiety and suicide.

## Data Availability

No datasets were generated or analysed during the current study.
